# The Effect of Different Concentrations of Chlorhexidine Gluconate on the Compressive Strength of Mineral Trioxide Aggregate

**DOI:** 10.15171/joddd.2015.001

**Published:** 2015-03-04

**Authors:** Maryam Bidar, Neda Eslami, Neda Naghavi, Zohreh Fasihi, Negin Attaran Mashhadi

**Affiliations:** ^1^Professor of Endodontics, Dental Research Center, Faculty of Dentistry, Mashhad University of Medical Sciences, Mashhad, Iran; ^2^Assistant Professor of Orthodontics, Dental Research Center, Faculty of Dentistry, Mashhad University of Medical Sciences, Mashhad, Iran; ^3^Assistant Professor of Endodontics, Dental Research Center, Faculty of Dentistry, Mashhad University of Medical Sciences, Mashhad, Iran; ^4^Dentist, Private Practice, Mashhad, Iran; ^5^Student of Medicine, Member of Student Research Committee, Faculty of Medicine, Mashhad University of Medical Sciences, Mashhad, Iran

**Keywords:** Chlorhexidine, compressive strength, Mineral Trioxide Aggregate

## Abstract

***Background and aims.*** Substituting chlorhexidine (CHX) for water has been shown to enhance antimicrobial activity of mineral trioxide aggregate (MTA). The purpose of this study was to compare the compressive strength of MTA mixed with distilled water, 0.12% and 0.2% chlorhexidine.

*** Materials and methods.*** MTA was mixed according to manufacturer's instructions in group I (n = 20). In groups II & III, 0.12% and 0.2% CHX liquid was substituted for water, respectively. Samples were condensed with moderate force into 20 tubes with 1.5×5 mm dimensions and were allowed to set for 72 hours at 37°C in 100% humidity. After being removed from the molds, their compressive strength was determined using Instron testing machine. Each group was divided into two subgroups according to the time of testing (at 72 hours, and one week). Fractured surfaces of 4 specimens in each group were then evaluated under Scanning Electron Microscope (SEM) to determine their microstructure. One-way ANOVA, Tukey, and paired sample t-test was used for statistical analysis. P < 0.05 was set as significant.

***Results.*** There was no significant difference between three groups in terms of their compressive strength after 72 hours. However, the compressive strength of group II was significantly higher than group I (P = 0.034) and group III (P = 0.021) after one week. Crystalline microstructure was similar in all groups.

***Conclusion.*** Substitution of 0.012% chlorhexidine for water significantly increased the compressive strength of MTA at 1 week without significant change in crystalline structure.

## Introduction

Since its first introduction to dentistry in 1993, mineral trioxide aggregate (MTA) has been widely used as the material of choice for some procedures. MTA shows better sealing ability compared to amalgam, IRM or super EBA,^1,2^ and is therefore used for sealing and repairing root perforations,^3,4^ and forming apical barrier.^5^ It is also applied as a root-end filling material.^6^ Furthermore, some studies have shown less cytotoxicity of MTA compared to amalgam, IRM and super EBA.^7^ Torabinejad et al^1^ reported that the compressive strength of MTA is significantly less than that of amalgam, IRM or super EBA after 24 hours. However, they found no significant difference in compressive strength of the three materials after three weeks. Another recent experiment revealed that keeping white MTA in dry conditions decreases its compressive strength.^8^ In general, the compressive strength of MTA is not affected by condensation pressure.^9^ Several factors can influence the compressive strength including the type of MTA, the liquid that is mixed with the material, pH of the mixing liquid, and the condition of MTA storage.^10,11^

Chlorhexidine gluconate (CHX) is an effective antimicrobial agent that acts against gram-positive and gram-negative bacteria, viruses, moulds, and facultative anaerobes and aerobes. Its mechanism of action is explained by damage to the cell wall and causing leakage of intracellular components and eventual death of the microorganisms.^12^ CHX mouthrinse (0.12%) can prevent the formation of plaque and decrease gingivitis.^13^ Substituting 0.12 % CHX for water enhances the antimicrobial activity of tooth colored ProRoot MTA for endodontic procedures.^14-16^ Alteration in the sealing properties of MTA when mixed with CHX has not been observed.^17^ MTA/CHX has been reported to be a biocompatible mixture which can be well tolerated.^18^

However, there are conflicting results on the compressive strength of MTA/CHX mixture. Decreased compressive strength of MTA/CHX compared to MTA mixed with water has been reported previously.^19^ The purpose of this study was to compare the compressive strength of MTA mixed with distilled water, 0.12% and 0.2% chlorhexidine.

## Materials and Methods

In this *in vitro* study, 60 samples of ProRoot MTA (Dentsply Tulsa Dental Specialties, Tulsa, USA) were divided to three groups. In group I, ProRoot MTA (Dentsply Tulsa Dental Specialties, Tulsa, USA) was mixed with a spatula on a glass slab according to the manufacturer’s instructions. Samples in group II & III were mixed in the same manner, substituting 0.12% or 0.2% CHX liquid for water, respectively. In each group, 20 samples were condensed with moderate force by using a small plugger into 1.5 × 5 mm dimension tubes.

A glass slide covered one end of the tubes, and wrapped with a damp gauze sponge. Samples were allowed to set for 72 hours at 37°C in 100% humidity.

Then, they were removed from the molds and were placed lengthwise between the plates of Instron testing machine (Model 1125, Instron Corp., Norwood, USA) to determine their compressive strength. The load was applied in the long axis of the specimen. Samples in each group were divided into two subgroups according to the time of testing (at 72 hours, and one week). Samples were compressed at a rate of 1 mm/min, and maximum load required to fracture each specimen was recorded in mega pascals (MPa).

Fractured surface of 4 specimens in each group was then evaluated under Scanning Electron Microscope (Philips PSEM 500 ×, Eindhoven, The Netherlands) to determine their microstructure and morphology.

One-way ANOVA and Tukey test were used to determine any statistical differences in compressive strengths between three groups in each time interval. Also, paired sample *t*-test was used to compare the compressive strength of each subgroup after 72 hours with 1 week. P < 0.05 was set as statistically significant.

## Results

Table 1 shows mean compressive strength of three groups at 72 hours and 1 week.

**Table 1:** Mean (± SD) compressive strength of the studied groups after 72 hours and 1 week

	Compressive strength (MPa)			
Time	Group I (Water)	Group II (0.12% chlorhexidine)	Group III (0.2% chlorhexidine)	P value
72 hours	12.94 ± 3.26	15.21 ± 2.18	14.97 ± 4.16	0.511
1 week	25.47 ± 5.14	42.91 ± 1.02	23.75 ± 1.19	0.015

ANOVA test did not reveal significant differences between three groups in terms of their compressive strength after 72 hours. However, a significant difference was found between groups after 1 week.

According to Tukey test, the compressive strength of group II was significantly higher than group I (P = 0.034) and group III (P = 0.021) after one week.

According to the results of paired sample t-test, there was a significant increase in mean compressive strength of all three groups after one week (P < 0.001).

**Figure 1.**
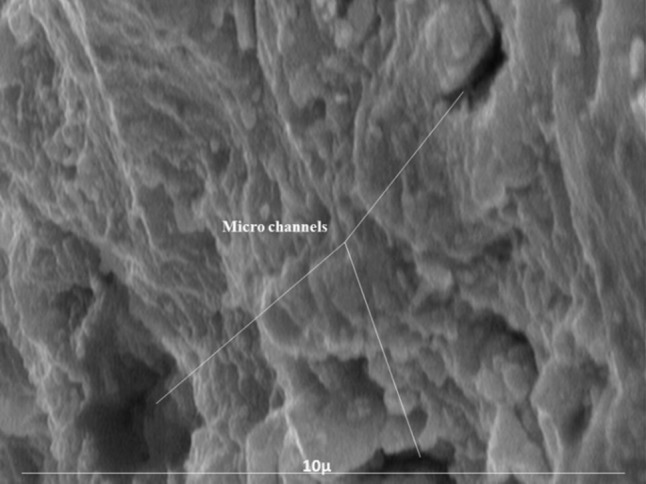
Crystalline microstructure after 72 hours of setting (SEM). No crystalline microstructure is seen in matrix.

**Figure 2.**
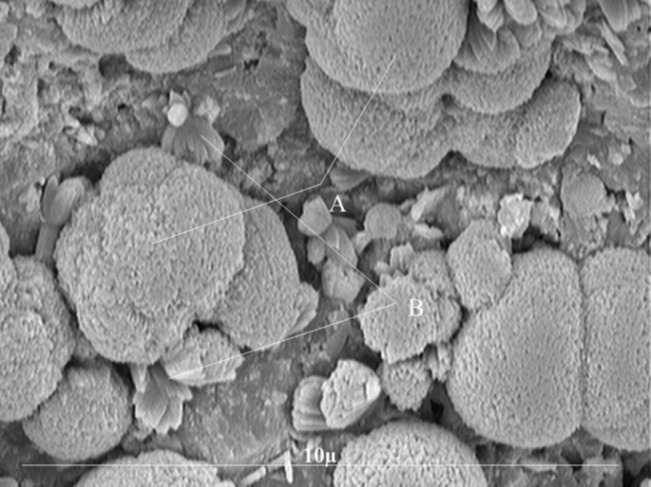
Crystalline microstructure after one week of setting (SEM). Large crystals with rounded edges embedded in globular matrix (A); Needle-like acicular crystals (B).

Results of SEM evaluation revealed that all three groups had roughly the same amorphous microstructure at 72 hours (Figure 1). However, large spherical forms with rounded edges embedded in globular matrix as well as needle-like acicular crystals could be seen in all groups after one week (Figure 2).

## Discussion

MTA has numerous applications since its introduction to endodontics. Several studies have reported appropriate sealing ability of MTA.^20,21^ Also, it has been shown that MTA is well tolerated by pulpal and periradicular tissues.^22^ Baek et al^23^ reported less inflammation with MTA than super EBA or amalgam. High biocompatibility of MTA is explained by its high pH.

MTA has been reported to be able to maintain a pH in the range of 11 to 12 for 78 days.^24^ Main et al^25^ observed a high rate of success after perforation repair with MTA for at least one year.

It has been shown that mixing MTA powder with chlorhexidine enhances its antimicrobial effects.^15,16,19^ In a previous study, no difference was found between various concentrations of CHX (0.12%, 0.2%, 2%) on the antimicrobial properties of MTA.^16^ However; mechanical properties should also be considered when substituting water for chlorhexidine. There is little evidence regarding compressive strength of MTA mixed with CHX.

In this study, MTA powder was combined with two concentrations of chlorhexidine to determine its compressive strength. MTA mixed with distilled water was used as the control in this study. The compressive strength of CHX groups was higher than that of the control group (12.94 MPa) after 72 hours of setting. However, the difference was not statistically significant (P > 0.05)

According to Torabinejad et al,^1^ the compressive strength of MTA after 24 hours was 40.0 MPA and increased to 67.3 MPa after 21 days. This difference can be attributed either to differences in methods of testing the compressive strength and/or changes in the composition of the MTA powder since it was first introduced. In the current study, the compressive strength of all three groups increased significantly at 7 days and was approximately doubled compared to that of 72 hours.

On the other hand, the compressive strength of MTA mixed with 0.12% CHX (42.91 MPa) was significantly higher than MTA mixed with 0.2% CHX (23.75 MPa) or control group (25.47 MPa) after 1 week. The compressive strength of MTA mixed with water was 28.4 MPa in the study of Kogan et al,^26^ which is similar to the results of the present study.

The importance of compressive strength of MTA varies according to its clinical application. When MTA is used for perforation repairs and additional forces are applied to the set material, high compressive strength of the MTA is required. Therefore, the clinician should either use a mixture with a higher compressive strength or place a barrier with a higher compressive strength.^26^

However, when MTA is used as a root-end filling material, reduced compressive strength is not considered as a major drawback due to minimal forces applied to retro-filling material.^26^ Considering the high compressive strength of 0.12% CHX / MTA, this mixture can be among the materials of choice for perforation repair or root-end fillings. However, other properties such as setting time and sealing abilities should be examined before its clinical application. Arrudaet al^17^ found that replacing distilled water with chlorhexidine did not alter the sealing properties of MTABio.

Contrary to our finding, Koganet al^26^ observed areas of MTA/CHX gel that were not completely set even after 7 days. This may indicate that mixing MTA powder with chlorhexidine liquid is a better alternative than chlorhexidine gel. Also, the difference in size of the specimens and sensitivity of the different testing apparatus may contribute to this discrepancy.

In addition to mechanical properties, biocompatibility of MTA/CHX mixture should also be considered. Should the addition of CHX to MTA compromise its biocompatibility, enhanced compressive strength and antimicrobial properties would be of no value. Sumer et al^18^ reported that MTA mixed CHX was surrounded by fibrous connective tissue in a rat model, which indicated that it was tolerated by the tissue. However, Hernandez et al^27^ demonstrated that substitution of 0.12% CHX for sterile water in MTA increased its cytotoxicity *in vitro*. However, the presence of serum *in vivo* may provide some protection against its cytotoxic effects. Faria et al^28^ proved that 0.25% CHX could cause small foci of tissue necrosis while 0.125% CHX resulted in no necrosis at all; although moderate inflammatory infiltrate was seen in both concentrations.

It seems that further *in vivo* investigations are required before routine use of the mixture of MTA with 0.12% chlorhexidine.

## Conclusion

When mixing MTA powder, substitution of 0.12% chlorhexidine for water significantly increased its compressive strength at 1 week without significantly changing its crystalline structure. With enhanced antimicrobial activity and less cytotoxicity of the mixture of MTA with 0.12% CHX verified previously, substituting 0.12% CHX for water in preparing ProRoot MTA mixture can be suggested.

## References

[1] Torabinejad M, Hong CU, McDonald F, Pitt Ford TR (1995). Physical and chemical properties of a new root-end filling material. J Endod.

[2] Fischer EJ, Arens DE, Miller CH (1998). Bacterial leakage of mineral trioxide aggregate as compared with zinc-free amalgam, intermediate restorative material and Super-EBA as a root-end filling material. J Endod.

[3] Chong BS, Pitt Ford TR, Watson TF, Wilson RF (1995). Sealing ability of potential retrograde root filling materials. Endod Dent Traumatol.

[4] Holland R, de Souza V, Nery MJ, Otoboni Filho JA, Bernabé PF, DezanJúnior E (1999). Reaction of dogs' teeth to root canal filling with mineral trioxide aggregate or a glass ionomer sealer. J Endod.

[5] Witherspoon DE, Ham K (2001). One-visit apexification: technique for inducing root-end barrier formation in apical closures. Pract Proced Aesthet Dent.

[6] Bates CF, Carnes DL, del Rio CE (1996). Longitudinal sealing ability of mineral trioxide aggregate as a root-end filling material. J Endod.

[7] Torabinejad M, Hong CU, Pitt Ford TR, Kettering JD (1995). Cytotoxicity of four root end filling materials. J Endod.

[8] Chogle S, Mickel AK, Chan DM, Huffaker K, Jones JJ (2007). Intracanal assessment of mineral trioxide aggregate setting and sealing properties. Gen Dent.

[9] Nekoofar MH, Adusei G, Sheykhrezae MS, Hayes SJ, Bryant ST, Dummer PM (2007). The effect of condensation pressure on selected physical properties of mineral trioxide aggregate. Int Endod J.

[10] Watts JD, Holt DM, Beeson TJ, Kirkpatrick TC, Rutledge RE (2007). Effects of pH and mixing agents on the temporal setting of tooth-colored and gray mineral trioxide aggregate. J Endod.

[11] Islam I, Chng HK, Yap AU (2006). Comparison of the physical and mechanical properties of MTA and portland cement. J Endod.

[12] Chate RA, White S, Hale LR, Howat AP, Bottomley J, Barnet-Lamb J (2006). The impact of clinical audit on antibiotic prescribing in general dental practice. Br Dent J.

[13] Yates R, Shearer BH, Huntington E, Addy M (2002). A method to compare four mouthrinses: time to gingivitis level as the primary outcome variable. J Clin Periodontol.

[14] Stowe TJ, Sedgley CM, Stowe B, Fenno JC (2004). The effects of chlorhexidine gluconate (012%) on the antimicrobial properties of tooth-colored ProRoot mineral trioxide aggregate. J Endod.

[15] Mittag SG, Eissner C, Zabel L, Wrbas KT, Kielbassa AM (2012). The influence of chlorhexidine on the antibacterial effects of MTA. Quintessence Int.

[16] Bidar M, Naderinasab M, Talati A, Ghazvini K, Asgari S, Hadizadeh B (2012). The effects of different concentrations of chlorhexidine gluconate on the antimicrobial properties of mineral trioxide aggregate and calcium enrich mixture. Dent Res J.

[17] Arruda RA, Cunha RS, Miguita KB, Silveira CF, De Martin AS, Pinheiro SL (2012). Sealing ability of mineral trioxide aggregate (MTA) combined with distilled water, chlorhexidine and doxycycline. J Oral Sci.

[18] Sumer M, Muglali M, Bodrumlu E, Guvenc T (2006). Reactions of connective tissue to amalgam, intermediate restorative material, mineral trioxide aggregate, and mineral trioxide aggregate mixed with chlorhexidine. J Endod.

[19] Holt DM, Watts JD, Beeson TJ, Kirkpatrick TC, Rutledge RE (2007). The anti-microbial effect against enterococcus faecalis and the compressive strength of two types of mineral trioxide aggregate mixed with sterile water or 2% chlorhexidine liquid. J Endod.

[20] Camilleri J (2009). Evaluation of selected properties of mineral trioxide aggregate sealer cement. J Endod.

[21] Post LK, Lima FG, Xavier CB, Demarco FF, Gerhardt-Oliveira M (2010). Sealing ability of MTA and amalgam in different root-end preparations and resection bevel angles: an in vitro evaluation using marginal dye leakage. Braz Dent J.

[22] Bidar M, Zarrabi MH, Tavakol JA, Aghasizadeh N, Naghavi N, Forghanirad M (2011). Osteoblastic cytokine response to gray and white mineral trioxide aggregate. Iran Endod J.

[23] Baek SH, Plenk H Jr, Kim S (2005). Periapical tissue responses and cementum regeneration with amalgam, SuperEBA, and MTA as root-end filling materials. J Endod.

[24] Fridland M, Rosado R (2003). Mineral trioxide aggregate (MTA) solubility and porosity with different water-to-powder ratios. J Endod.

[25] Main C, Mirzayan N, Shabahang S, Torabinejad M (2004). Repair of root perforations using mineral trioxide aggregate: a long-term study. J Endod.

[26] Kogan P, He J, Glickman GN, Watanabe I (2006). The effects of various additives on setting properties of MTA. J Endod.

[27] Hernandez EP, Botero TM, Mantellini MG, McDonald NJ, Nör JE (2005). Effect of ProRoot MTA mixed with chlorhexidine on apoptosis and cell cycle of fibroblasts and macrophages in vitro. Int Endod J.

[28] Faria G, Celes MR, Rossi AD, Silva JS, Silva LA, Rossi MA (2007). Evaluation of chlorhexidine toxicity injected in the paw of mice and added to cultured l929 fibroblasts. J Endod.

